# Validating the use of stereo‐video cameras to conduct remote measurements of sea turtles

**DOI:** 10.1002/ece3.7653

**Published:** 2021-05-26

**Authors:** Tabitha R. Siegfried, Mariana M. P. B. Fuentes, Matthew Ware, Nathan J. Robinson, Emma Roberto, Joseph R. Piacenza, Susan E. Piacenza

**Affiliations:** ^1^ University of West Florida Pensacola FL USA; ^2^ Department of Earth Ocean and Atmospheric Science Florida State University Tallahassee FL USA; ^3^ Fundacion Oceanogràfic Oceanogràfic de València Valencia Spain; ^4^ Cape Eleuthera Island School Cape Eleuthera Institute Eleuthera The Bahamas

**Keywords:** body size, green turtle, Kemp's Ridley turtle, loggerhead turtle, morphology, photogrammetry

## Abstract

Point 1: Stereo‐video camera systems (SVCSs) are a promising tool to remotely measure body size of wild animals without the need for animal handling. Here, we assessed the accuracy of SVCSs for measuring straight carapace length (SCL) of sea turtles.

Point 2: To achieve this, we hand captured and measured 63 juvenile, subadult, and adult sea turtles across three species: greens, *Chelonia mydas* (*n* = 52); loggerheads, *Caretta caretta* (*n* = 8); and Kemp's ridley, *Lepidochelys kempii* (*n* = 3) in the waters off Eleuthera, The Bahamas and Crystal River, Florida, USA, between May and November 2019. Upon release, we filmed these individuals with the SVCS. We performed photogrammetric analysis to extract stereo SCL measurements (eSCL), which were then compared to the (manual) capture measurements (mSCL).

Point 3: mSCL ranged from 25.9 to 89.2 cm, while eSCL ranged from 24.7 to 91.4 cm. Mean percent bias of eSCL ranged from −0.61% (±0.11 *SE*) to −4.46% (±0.31 *SE*) across all species and locations. We statistically analyzed potential drivers of measurement error, including distance of the turtle to the SVCS, turtle angle, image quality, turtle size, capture location, and species.

Point 4: Using a linear mixed effects model, we found that the distance between the turtle and the SVCS was the primary factor influencing measurement error. Our research suggests that stereo‐video technology enables high‐quality measurements of sea turtle body size collected in situ without the need for hand‐capturing individuals. This study contributes to the growing knowledge base that SVCS are accurate for body size measurements independent of taxonomic clade.

## Graphical abstract

Are stereo‐video cameras accurate for marine megafauna? This new study suggests that SVC are highly accurate for sea turtles (mean percent bias −0.76%–4.46% across species). Accuracy was influenced by distance between the SVC and the turtles, but not by angle, image quality, or animal size.

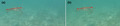

## 1 INTRODUCTION

An animal's morphology can provide valuable insights into its ecology and behavior (Lutz et al., 1996; Webb, 1984; White et al., 2007). Collecting morphological measurements can be achieved either by hand (i.e., direct measurements) or remotely (i.e., indirect measurements). While direct measurements are generally highly accurate, they often require animals to be captured, handled, and restrained. Handling of animals can provoke a stress‐response, and it often has inherent risks for both the animal and the researcher (Harcourt et al., 2010; Omsjoe et al., 2009; Schofield et al., 2008). In contrast, indirect methods often avoid these issues, yet they require validation to determine their accuracy. One method for obtaining remote body size measurements is using a stereo‐video camera system (SVCS; Harvey et al., 2002; Harvey & Shortis, 1998; Shafait et al., 2017).

Stereo‐video camera systems use two cameras from overlapping perspectives to create a three‐dimensional image from which measurements can be extracted using the principle of collinearity (Harvey & Shortis, 1996). Collinearity is the geometric concept that three points of interest will be in alignment. Here, the three points of interest are the central projection from the camera, the focal plane of the camera, and the image point (Harvey & Shortis, 1996). SVCS have accurately measured sharks (Delacy et al., 2017), southern bluefin tuna (Harvey et al., 2003), cetaceans (Hillcoat et al., 2020; Spitz et al., 2000), and a variety of fish species (e.g., Davis et al., 2015; Dunbrack, 2006). However, these studies have been conducted on animals with a flexible body that swim by contracting and relaxing muscles laterally in a sinusoidal pattern, which can lead to variability between individual measurements (Harvey et al., 2003). Species with stationary and rigid points of measurements, such as the carapace of sea turtles, might provide more reliable and accurate reference points for SVCS measurements. While SVCS measurements of sea turtles have been compared to visual estimates and laser photogrammetry (Araujo et al., 2019), they have not yet been compared to direct hand measurements.

Body size data of all life stages of sea turtles are fundamental to understanding population structure (e.g., gender and size‐class distribution). Further knowledge of body size measurements is needed to properly assign individuals to specific life stages, and to estimate somatic growth rates, age‐at‐maturity, and survival rates (Bjorndal et al., 2000; Casale et al., 2011; Quinn & Deriso, 1999). Body size data of sea turtles are often collected in different ways depending on the targeted life stage: nesting females are measured during nesting surveys, and broad population structure is obtained from stranded sea turtles that have been rescued (Avens et al., 2017; Scherer et al., 2014; Vélez‐Rubio et al., 2013) or from turtles that have been captured in‐water using hand or net capturing methods (Fuentes et al., 2006; Limpus & Walter, 1980; Schofield et al., 2008; Wildermann et al., 2019). Implementing the use of SVCS could potentially increase sample size across life stages and, therefore, minimize inherent bias in the sampled population.

Here, we assessed the practicality of using a SVCS to provide accurate measurements of body size in sea turtles. To achieve this, we compared direct measurements of straight carapace length (SCL) in three different sea turtle species (greens, loggerheads, and Kemp's ridley*)* to remote measurements from a SVCS. By investigating three different sea turtle species, each of which has distinct shell morphologies, but relatively consistent points of measurement, we were able to investigate inter‐species effects on the accuracy of SVCS. Finally, we investigated potential factors that may influence the accuracy of SVCS for measuring sea turtles, including the object distance to SVCS, image quality, individual size, species, capture location, and turtle orientation.

## 2 MATERIALS & METHODS

### 2.1 Study site and capture of turtles

**FIGURE 1:**
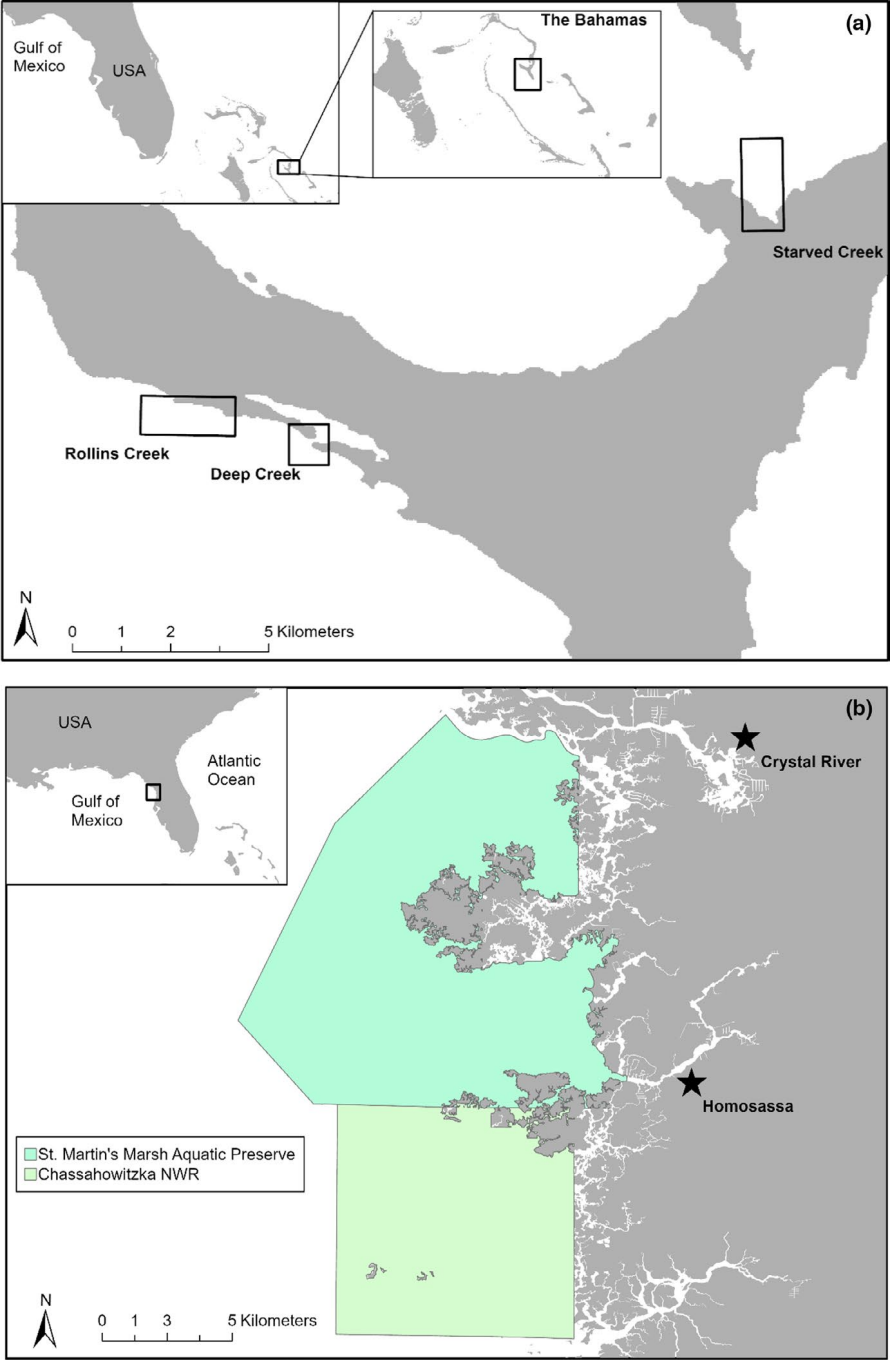
Study sites of the two regions where turtles were captured and filmed. Cape Eleuthera, Bahamas, sea turtles were captured at three locations: Rollins Creek, Deep Creek, and Starved Creek (a). Crystal River, FL, sea turtles were captured within the two protected areas of St. Martin's Marsh Aquatic Preserve and Chassahowitzka National Wildlife Refuge located in Crystal River and the northwest region of Chassahowitzka (b)

This study was conducted in mangrove creeks off Eleuthera, The Bahamas and in seagrass beds off Crystal River, Florida, USA (Figure 1). During a 2‐week period in July 2019, we captured turtles in Eleuthera using a modified “rodeo” technique. Specifically, we briefly followed the turtles by boat before capturing them on snorkel. In total, we captured and successfully measured 48 unique individuals. In Crystal River, we conducted surveys between May and November 2019, and successfully captured and measured 15 individual turtles using traditional rodeo and dip‐netting techniques (Fuentes et al., 2006; Limpus & Walter, 1980).

### 2.2 Handling of turtles

Captured turtles were brought onboard the research boat for direct measurements. SCL was measured between the anterior point on the nuchal scute to the posterior tip of the supracaudal scutes along the midline (±0.1 cm; SCL) with metal calipers (Bolten, 1999). Turtles were then tagged using either metal flipper tags or Passive Integrated Transponders (PIT tag, Biomark, GPT12), if they did not already have tags, before being released.

### 2.3 Stereo‐video camera calibration

The SVCS was comprised of a SeaGIS underwater camera housing designed to hold two cameras (Go Pro Hero 5 Black) at a fixed distance apart (0.8 m). The cameras were inwardly aimed at an angle of ~4°. Each camera was set at 1,920 × 1,080 pixel video resolution, medium field of view, and 30 frames/sec. The SVCS was calibrated at the start and conclusion of each field season at the University of West Florida Aquatic Center in <1 m depth, using the method described by Shortis and Harvey (1998). In short, the calibration used multiple images of a three‐dimensional aluminum cuboid frame marked with 56 precisely known reference points. The locations of these points were measured in multiple images taken from 20 standardized orientations of the calibration cube. Images were analyzed in SeaGIS CAL software v.3.23 (SeaGIS EventMeasure, SeaGIS Pty, Bacchus Marsh, 2008) to calibrate both the internal and external parameters of the SVCS (Boutros et al., 2015). Calibration included calculating the camera parameters, including the focal lengths (i.e., the distance that the lens converges light) of the cameras. The focal length of the cameras was fixed parameters, with the left camera having a focal length of 3.89 mm, and the right camera 3.90 mm.

### 2.4 Stereo‐video camera deployment

Before commencing video data collection, we synchronized the two SVCS cameras using unique initial cues (e.g., hand clapping or touching fingers) at the beginning of the recording session. To record a free‐swimming turtle, we entered the water with the SVCS prior to a turtle's release. We released turtles from the boat in the general direction of the SVCS or off to the side to allow for adequate video footage to be obtained. In Eleuthera, we followed turtles with the SVCS until the turtle was no longer visible. In Crystal River, we did not pursue turtles due to permitting restrictions.

### 2.5 Comparison analysis

**FIGURE 2:**
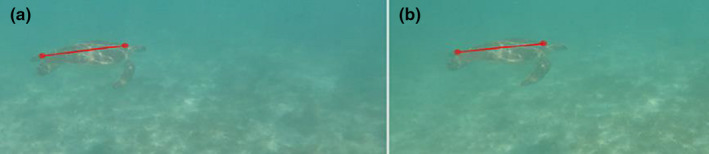
EventMeasure user interface illustrating paired images with sea turtle length measurement vectors illustrated in red in the left (a) and right (b) cameras

Measurements from the SVCS were extracted using EventMeasure software v.5.22 (SeaGIS Pty Ltd). From the image pairs, the same anterior and posterior points as described above were selected from the right and left cameras. SCL was calculated as the distance between these points (Figure 2a,b; Harvey et al., 2002). To reduce measurement error, we extracted stereo measurements (eSCL) in 10 image pairs from different video frames (Harvey et al., 2001).

We tested the accuracy of the SVCS to measure SCL in two ways. First, using the average of the 10 eSCL measurements (Harvey et al., 2001), we conducted a linear regression to estimate the slope of the relationship between the mean eSCL and manual capture measurements (mSCL). If the slope was equal to one, then this would suggest a perfect agreement in the two measurement types. We conducted initial diagnostic tests to ascertain whether the residuals of the data conformed to the assumptions of a linear model (i.e., were normally distributed and homoscedastic). We found the assumptions were upheld based on visual inspection of the quantile‐quantile plots, the fitted values and the residuals, and a frequency distribution of the residuals. We then computed the Akaike information criterion correction for small sample sizes (AICc) of each linear model for each species, across both sites, and compared it to the AICc of the null intercept‐only model for each species. Next, using all 10 individual measurements from each turtle, we assessed the directionality of the relationship between eSCL and mSCL by calculating percent bias. We calculated percent bias using the following equation:

(1) $$\delta_{1}=100\%\times \frac{v‐v_{\text{est}}}{v}$$

where *δ*_1_ was the percent bias, *v* was the mSCL measured by hand with calipers, and *v*_est_ was eSCL from the stereo measurements (Table 1).

**TABLE 1:** AICc model values for linear model comparing the eSCL to the mSCL

Model	Slope	AICc	Null AICc (eSCL = 1)	Average percent bias
Greens	0.98 ± 0.01 *SE*	156.24	389.02	−0.61% ± 0.11 *SE*
Loggerheads	1.08 ± 0.07 *SE*	43.34	67.14	−0.76% ± 0.30 *SE*
Kemp's ridley	0.84 ± 0.03 *SE*	AIC = 3.017	AIC = 21.64	−4.46% ± 0.31 *SE*

Note

AICc = Akaike's Information Criterion corrected for small sample sizes. Null AICc values are for the null models (eSCL=1) for each species. AIC was calculated for Kemp's ridley sea turtles because AICc was unable to be calculated due to small sample size. Slope is from the best fit line for all linear models. Average percent bias of all measurements for each species.

### 2.6 Evaluating factors influencing measurement accuracy

To evaluate how various factors influence the accuracy of eSCL, we used the 10 individual measurements from each turtle to calculate percent error. We used the measurements from all 10 image pairs because each measurement had a different value for the factors of interest (e.g., distance from the turtle to SVCS (range), image quality, and angle). Thus, the parameters in the global model were at the scale of the measurement, not the individual turtle. Therefore, this resulted in an overall sample size of *n* = 630, with 520 green, 80 loggerhead, and 30 Kemp's ridley sea turtles. Percent error was calculated as:

(2) $$\delta_{2}=100\%\times \left|\frac{v‐v_{\text{est}}}{v}\right|$$

where *δ*_2_ was the percent error, *v* was the mSCL measured by hand with calipers, and *v*_est_ was eSCL from the stereo measurements. Percent error (or the absolute value of the percent bias) was used instead of percent bias in this analysis because we were not concerned with the directionality of the relationship. Percent error allowed for clearly distinguished patterns of the relationship with the potential explanatory variables and percent error regardless of the direction of bias.

We compared percent error to distance from the turtle to the SVCS (range), turtle orientation relative to the SVCS (angle), image quality, size of turtle, capture location, and species observed, in a log‐transformed linear regression model. Testing of the residuals of the untransformed global model displayed heterogeneity in variance based on visual inspections of the residuals to fitted values, frequency distribution of residuals, and residuals plotted against the explanatory variables, so the response variable, percent error, was log‐transformed (Zuur et al., 2009). As turtles were repeatedly measured for the eSCL, we included a repeated effect for each individual turtle. Therefore, we ultimately evaluated factors influencing the log‐transformed percent error using a linear mixed effect model (LMM), using the R package *lme4* (Bates et al., 2015), which substantially improved model fit, based on AICc.

Range (m), or the distance of the turtle from the SVCS, was automatically calculated within the EventMeasure software and was reported when measurements were extracted. For turtle orientation, we quantified different orientations of the turtle relative to the camera system. Here, we visually estimated the angles ranging from 0° to 360°, at increments of 45°. An angle of 0° represented the turtle swimming away from the diver, while 90° represented the turtle swimming in front of the camera heading right. Image quality was assigned to each measurement frame on a scale of 1–3 based on the clarity of the posterior and anterior carapace points. If the two points were easily distinguished, then the frame was given a photo quality rating of 1. If the two points were more difficult to distinguish but visible, the frame was given a photo quality rating of 2. If the two points were unclear but could be interpolated, the frame was given a photo quality rating of 3. We tested the numeric explanatory variables for collinearity using variance inflation factors (VIFs) and all had scores <10, our a priori threshold (Zuur et al., 2009).

To identify which explanatory variables affected percent error, we used the information theoretic approach for model selection, using AICc (Burnham & Anderson, 2002; Johnson & Omland, 2004), with the dredge function in the R package *MuMIn* (Barton, 2020). Models with ΔAICc < 2 from the top‐ranked model were retained in the confidence model set. If multiple models were retained in the confidence set, we examined the relative importance of each variable included in the confidence model set based on the sum weight of all models containing that variable (Burnham & Anderson, 2002). High values of relative importance results from variables occurring in a large proportion of the confidence model set. Further, we investigated if the explanatory variables included in the confidence set were uninformative, that is, had confidence intervals that crossed zero (Arnold, 2010; Burnham & Anderson, 2002; Leroux, 2019). Residuals from the top‐ranked model also provided a supplementary method to visually assess model assumptions and fit. All candidate models were tested against our global model:

(3) $$\text{ln}\;\left({\text{Percent error}}_{i}\right)=\beta_{0}+\beta_{1}\times \text{Range}+\beta_{2}\times \text{Image Quality}+\beta_{3}\times \text{Angle}+\beta_{4}\times \text{SCL}+\beta_{5}\times \text{Species}+\beta_{6}\times \text{Location}+a_{i}+\varepsilon_{i,j}$$

where *a_i_* ~ N(0, *σ* ^2^_turtle_ID_), and *ε_i_* ~ *N*(0, *σ* ^2^) of turtle *i*. All analyses were performed in R v.3.5.2 (R.C. Team, 2018 ) and R Studio v.1.0.153 (Rstudio Team, 2020).

## 3 RESULTS

### 3.1 Stereo‐video verification

**FIGURE 3:**
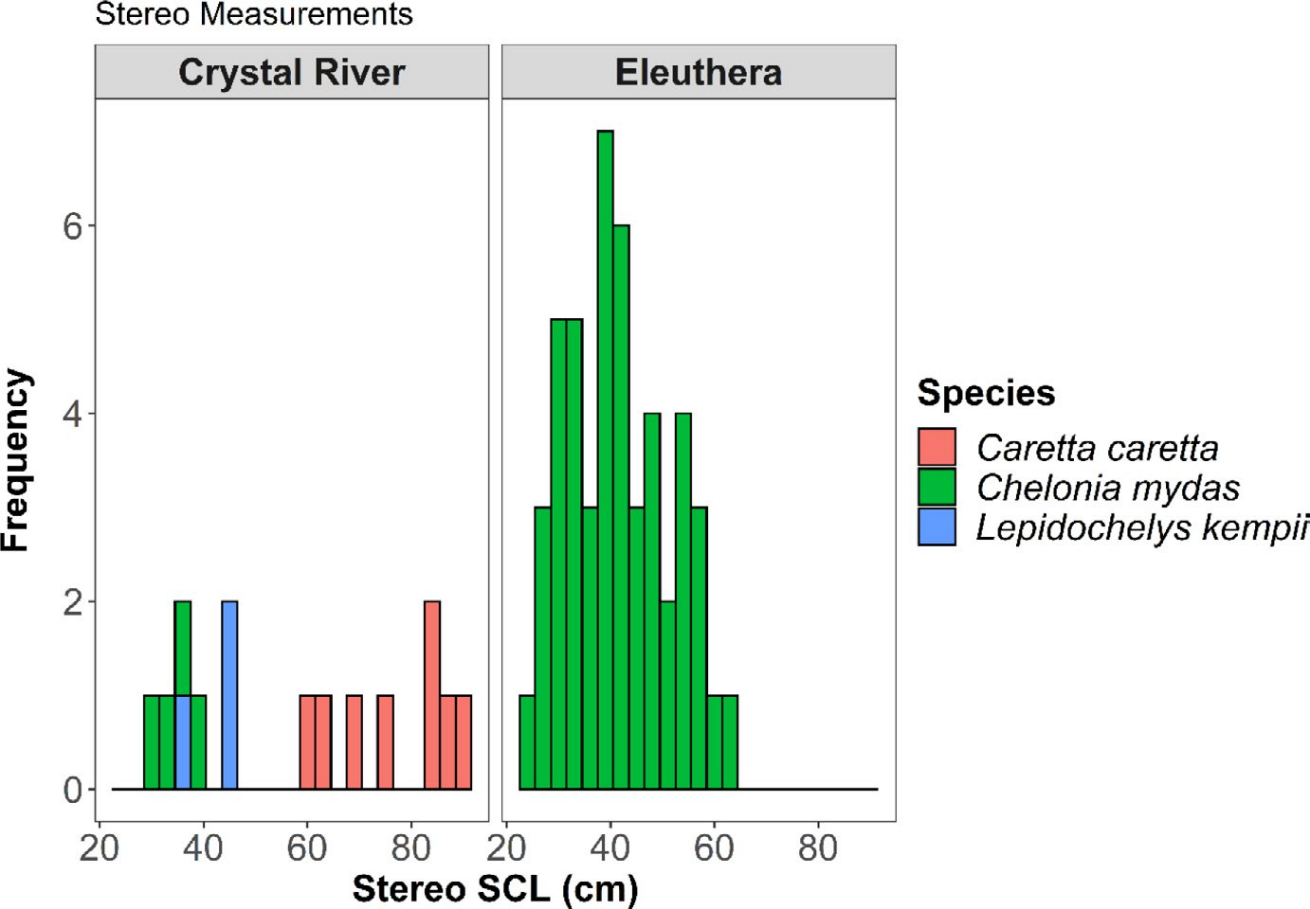
Frequencies of the stereo‐video‐derived straight carapace length (eSCL) of three species: green, loggerhead, and Kemp's ridley sea turtles at Eleuthera, Bahamas and Crystal River, Florida, USA

We analyzed 63 sea turtles (green [*n* = 52], loggerhead [*n* = 8], and Kemp's ridley [*n* = 3]) in EventMeasure to obtain eSCL through SVCS. All turtles (*n* = 48) in Eleuthera were green sea turtles and mSCL ranged from 25.9 to 63.7 cm with a mean length of 42.9 (±10.5 *SD*) cm mSCL. In Crystal River, loggerhead sea turtles (*n* = 8) mSCL ranged from 60.6 to 89.2 cm with a mean length of 76.8 (±10.5 *SD*) cm mSCL, green sea turtles (*n* = 4) mSCL ranged from 30.5 to 37.2 cm with a mean length of 34.2 (±2.8 *SD*) cm mSCL, and Kemp's ridley sea turtles (*n* = 3) mSCL ranged from 35.5 to 47.8 cm with a mean length of 43.2 (±6.7 *SD*) cm mSCL (Figure 3).

**FIGURE 4:**
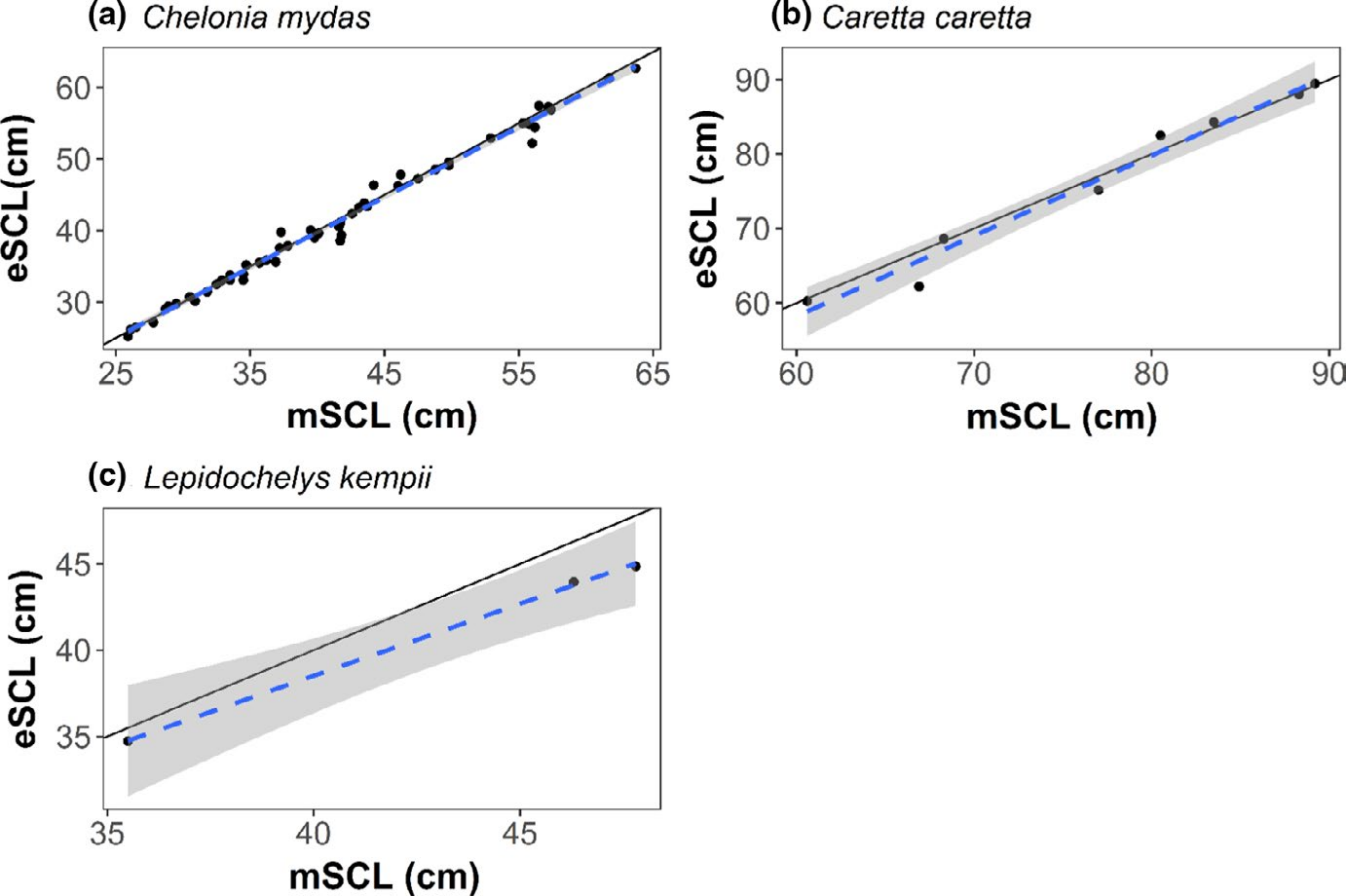
Relationship between SCL measured by hand (mSCL) and the SCL measured by the stereo‐video camera system (eSCL) for green (a), loggerhead (b), and Kemp's ridley (c) sea turtles. The solid black line is a diagonal one‐to‐one line, and the dotted lines are the linear model (LM) predicted fit with the shading for the 95% confidence interval. The LM slope was estimated to be 0.98 (±0.01 *SE*) for green, 1.08 (±0.07 *SE*) for loggerhead, and 0.84 (±0.03 *SE*) for Kemp's ridley sea turtles

When comparing mSCL and average eSCL via linear regression, the slope varied based on species: 0.98 (±0.01 *SE*) for greens, 1.08 (±0.07 *SE*) for loggerhead, and 0.84 (±0.03 *SE*) for Kemp's ridley sea turtles (Figure 4a,c). Each model had lower AICc values than the null model for each species, and the null models were well outside ΔAICc < 2 (Table 1). For Kemp's ridley sea turtles, we used AIC as AICc could not be calculated due to the sample size.

Percent bias in eSCL measurements varied by species; however, eSCL measurements were always underestimated. For each species, percent bias was as follows: −0.61% (±0.11 *SE*) for green (*n* = 52), −0.76% (±0.30 *SE*) for loggerhead (*n* = 8), and −4.46% (±0.31 *SE*) for Kemp's ridley (*n* = 3) sea turtles.

### 3.2 Factors influencing accuracy

**FIGURE 5:**
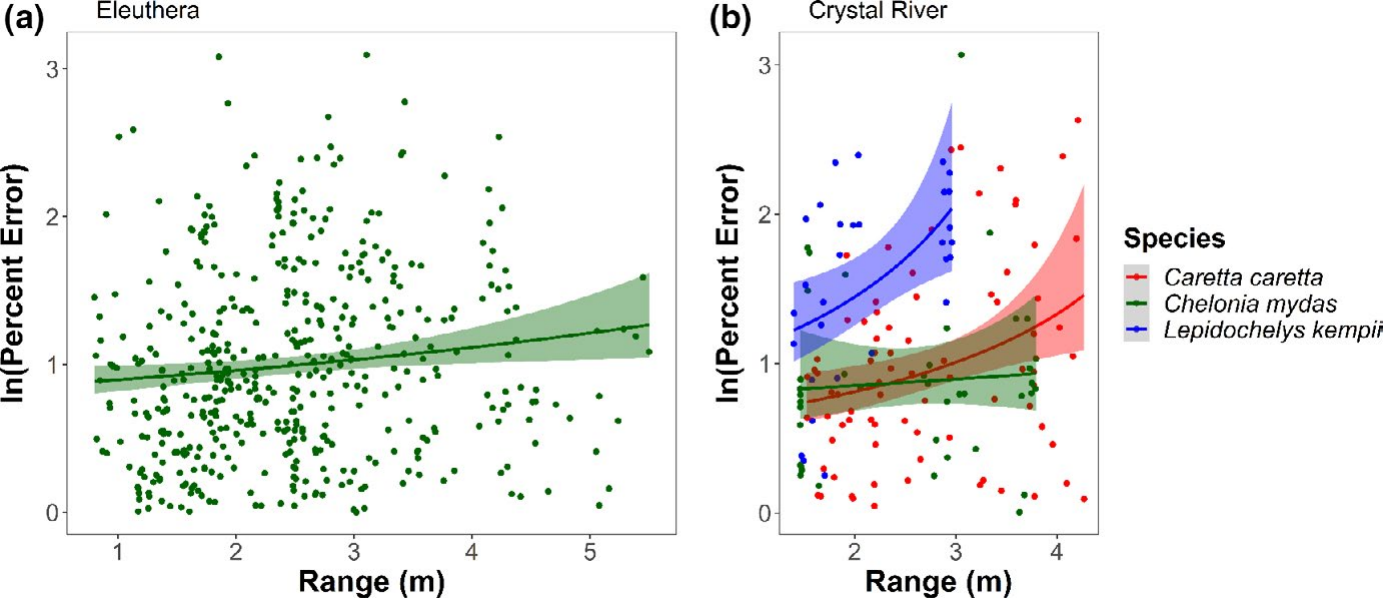
Comparison of log‐transformed percent error to range of the turtle from the cameras with linear mixed model (LMM) predictions. The lines are color coded by species and represent the LMM predicted fit with shading for the 95% confidence interval. Green sea turtles in Eleuthera (a) and the three species in Crystal River (b)

When evaluating the effect of angle, image quality, species, body size, location, and the distance between the SVCS and the turtle on the accuracy of the eSCL measurements, the model confidence set included two top‐ranked models, which both included range and species (Table 2, see Appendix 1 for full model selection table). The evidence ratio of the top‐ranked model compared to the intercept‐only model was 29.55; this suggests that the top‐ranked model was 29 times more likely to occur than the null model. As the range of the sea turtles from the SVCS increased, the percent error of the eSCL measurements increased, but this varied by species and location (Figure 5). Species was included in the second model in the confidence set and has a relative importance of 0.47. Notably, orientation, size of the individual turtle, and image quality were not included in the confidence set of models.

**TABLE 2:** Confidence set for the linear mixed model prediction of ln‐percent error and potential explanatory variables (ΔAICc < 2)

Model Terms						Model Support				
	Angle	Image quality	eSCL	Location	Range	Species	*df*	AICc	ΔAICc	Weight
Model 1	–	–	–	–	0.17 (0.8–0.27)	−	4	1,078.40	0	0.53
Model 2	–	–	–	–	0.18	+	6	1,078.67	0.30	0.47
R.I.	–	–	–	–	1	0.47				

Note

95% confidence intervals provided in parentheses.

Abbreviations: −, explanatory variables not included in the model selection; +, explanatory variables included in the model selection; AICc, Akaike's Information Criterion corrected for small sample size; *df*, degrees of freedom; R.I., relative importance; ΔAICc, difference in AICc from the top ranked model and model in consideration.

## 4 DISCUSSION

Stereo‐video camera systems are an accurate and reliable tool for obtaining morphometric data for in‐water life stages (e.g., juvenile, subadult, and adult) of sea turtles. Low average percent bias was found in our study, ranging from −0.61% (±0.11 *SE*) to −4.46% (±0.31 *SE*) across all species, and the global average across all species was −0.99% (±0.01 *SE*). Our results are comparable to the mean error of 1.16% (range: −2.33%–3.00%) when using SVCS to measure oceanic whitetip sharks *Carcharhinus* *longimanus* (Delacy et al., 2017). However, it should be noted that Delacy et al. (2017) measured sharks while they were held taut against the side of the boat, rather than measuring them free‐swimming. Therefore, the error Delacy et al. (2017) estimated may be lower than that would be expected when measuring actively swimming elasmobranchs. Notably, observation error also occurs when collecting body size data via hand measurements. For example, using data from a mark–recapture program of juvenile greens in Cape Eleuthera, Bahamas, the mean percent error between measurements taken on the same individual less than 30 days apart (to avoid differences due to body growth) was 0.89% ± 0.099 *SE* (range: 0%–8.13%, *n* = 96; N. J. Robinson, unpubl. data). Therefore, the eSCL measurements fell well within the same range as the background level of observation error from traditional hand measurements.

The range, or distance between the SVCS and the target, appears to be the main factor influencing the accuracy of the SVCS. Our results suggest that as the distance between the turtle and the camera increases, accuracy of the eSCL measurements decreases. In Eleuthera, the percent error tended to increase after a 5 m distance between individual turtles and the SVCS. In Crystal River, percent error increased around 2 m for Kemp's ridley and 3 m for loggerhead sea turtles, whereas green sea turtles were relatively stable and within the range of measurements achieved in Eleuthera (<4 m). Therefore, it is advantageous to be closer to the sea turtle for accurate measurements. However, if the SVCS is too close to the sea turtle (<1 m), the turtle will not appear in both cameras and the video will be unusable. In general, the optimal range for the SVCS used here, which had a 0.8‐m separation between cameras, is between 2 and 8 m, with accuracy and precision diminishing around 8 m (Harvey et al., 2010). Notably, SVCS with different separation distances will have different optimal ranges.

Based on the model selection, image quality did not strongly influence percent error. This is encouraging as it indicates that it is not necessary to have a perfectly clear image to accurately measure body size. However, our study was conducted in the water off Eleuthera, Bahamas and Crystal River, Florida, where water visibility was relatively high and consistent, and resulted in clear images. According to Harvey et al. (2001), if the researcher can see the points of measurement or visually interpolate where they are, the length measurement will be accurate. Therefore, our study concurs with Harvey et al. (2001) in that image quality has little influence on the accuracy of stereo measurements. However, when visibility is low, for example, ≤2 m, the range between the SVCS and the sea turtle will need to be closer to 2 m, to ensure the turtle is distinguishable on the footage. Thus, image quality, as it relates to visibility, is more important for turtle detection, rather than influential to measurement accuracy.

The species of sea turtle appears to have an influence on measurement accuracy. While the inclusion of species in the model greatly improved the model fit and appears to be an informative parameter (i.e., the confidence interval does not cross zero), there are some additional considerations. First, the sample size used for analysis for both loggerheads (*n* = 8) and Kemp's (*n* = 3) was low in comparison with the sample size used for greens (*n* = 52). This could have influenced the selection for “species” in the model. Second, we found that percent error for Kemp's ridley and loggerheads in Crystal River had a greater rate of increase in relationship to range (Figure 5). This is potentially because the larger the object to measure, the greater the range needs to be for the turtle to be in the camera's field of view, especially for the adult loggerheads (Hillcoat et al., 2020). This was probably influenced by a learning bias as all Crystal River data were collected at the beginning of our study. Initially, ideal diver position in the water had not yet been determined to result in the best video footage for analysis. This resulted in limited useable frames for the larger individuals, as these individuals did not always co‐occur in the right and left cameras' field of views. In addition, there were some minor differences in sample collection between Crystal River and Eleuthera that may have also contributed to species‐level differences. In Crystal River, where all the loggerhead and Kemp's ridley sea turtles were collected, the water depth was slightly shallower (<2 m), release points were over seagrass beds, and following the animals was not permitted, which also resulted in limited useable video footage. In contrast, in Eleuthera, turtles were followed resulting in ample footage to analyze, in depths >3 m, and over a sandy bottom (which tended to improve image contrast and minimized turbidity from both the researcher and the turtle that occurred in the seagrass beds).

Ultimately, SVCSs are advantageous over other forms of in‐water morphometric methods, such as underwater visual surveys and laser photogrammetry, and comparable to direct measurements via hand capture. In addition to their high accuracy, the ability of SVCS to archive and re‐evaluate data is a benefit to researchers (Davis et al., 2015). In comparison with laser photogrammetry, SVCS photogrammetry is fundamentally more accurate due to its use of paired cameras (Webster et al., 2010). Laser photogrammetry uses a single camera with a pair of parallel lasers (Deakos, 2010; Perry et al., 2018; Rizzo et al., 2017) and accuracy measurements range from 0.39% to 5.2% (Deakos, 2010; Rogers et al., 2017; Trobbiani et al., 2018). In comparison, our error measurements ranged from −0.61% (±0.11 *SE*) to −4.46% (±0.31 *SE*). Laser photogrammetry has similar benefits to the SVCS in that it is an indirect method to collect body size measurements and data are archivable and can be reprocessed if desired. However, SVCS photogrammetry has the additional advantage because the individuals do not have to be in a perfect perpendicular field of view relative to the camera, which is necessary for laser photogrammetry (Perry et al., 2018; Rogers et al., 2017). While some SVCSs are more expensive than laser photogrammetry, it is possible to build a SVCS independently and use open‐source software, for example, VidSync, and freely available packages for R, to extract eSCL measurements (Boutros et al., 2015; Goetze et al., 2019).

Moving forward, SVCS could couple morphometric data with in‐water observations of behavior and habitat use of species. In addition, SVCS can be paired with photo identification to track individuals overtime, creating a remote mark–recapture dataset that when combined with traditional turtle capture studies would ultimately result in overall larger sampling of the wild population.

In this study, we have verified that SVCSs provide an accurate and reliable way to measure sea turtle body length remotely. This study contributes to a growing number of studies that have validated the accuracy of SVCS for body length measurement independent of taxonomic clade. As such, researchers will be able to conduct in‐water surveys to collect body size measurements of a variety of marine species, while reducing safety concerns of capturing animals and subsequently increasing sample sizes for current ongoing monitoring programs. Further, though few studies exist on terrestrial species to date, there is a growing community of scientists using SVCS terrestrially. For example, SVCS have been used to study bat echolocation call intensity (Holderied et al., 2005), and 3D tracking of avian and bat flight tracks (de Margerie et al., 2015; Matzner et al., 2020). SVCS methods can be applied to other terrestrial species and are especially useful for endangered species where capture methods require euthanasia or destructive sampling. As we found that the increasing distance between the SVCS and the organism increased measurement error, there is probably a similar relationship for terrestrial animals, although the distance limitation may differ due to the differences in optics between air and water. Additional studies using SVCS to measure terrestrial animals would be of great value to the scientific community. The implications for SVCS for conservation management efforts are immense and show great promise in enhancing current data availability by yielding critical data that are currently lacking for many populations globally.

## CONFLICT OF INTEREST

The authors declare no conflict of interest.

## AUTHOR CONTRIBUTIONS

**Tabitha Siegfried:** Conceptualization (lead); Data curation (lead); Formal analysis (lead); Funding acquisition (lead); Investigation (lead); Methodology (lead); Validation (lead); Writing‐original draft (lead). **Mariana Fuentes:** Funding acquisition (equal); Resources (lead); Supervision (equal); Visualization (equal); Writing‐review & editing (equal). **Matthew Ware:** Data curation (equal); Funding acquisition (equal); Investigation (equal); Supervision (equal); Validation (equal); Visualization (equal); Writing‐review & editing (equal). **Nathan Jack Robinson:** Data curation (equal); Funding acquisition (equal); Investigation (equal); Methodology (equal); Supervision (equal); Validation (equal); Visualization (equal); Writing‐review & editing (equal). **Emma Roberto:** Data curation (equal); Investigation (equal); Methodology (equal); Writing‐review & editing (equal). **Joe Piacenza:** Investigation (supporting); Supervision (supporting); Writing‐review & editing (supporting). **Susan Piacenza:** Conceptualization (equal); Data curation (equal); Formal analysis (equal); Funding acquisition (equal); Investigation (equal); Methodology (equal); Project administration (lead); Resources (equal); Supervision (lead); Validation (equal); Visualization (equal); Writing‐review & editing (lead).

## Data Availability

We agree to archive the data associated with this manuscript should the manuscript be accepted. Data are achieved in Dryad. https://doi.org/10.5061/dryad.hhmgqnkgk

## APPENDIX 1

**TABLE A1:** Linear mixed model output of ln‐percent error and potential explanatory variables for all models evaluated

Model terms						Model support				
	Angle	Image quality	eSCL	Location	Range	Species	*df*	AICc	ΔAICc	Weight
Model 1	–	–	–	−	0.17 (0.8–0.27)	−	4	1,078.40	0	0.30
Model 2	–	–	–	−	0.18	+	6	1,078.67	0.30	0.26
Model 3	–	0.10	–	−	0.15	+	7	1,080.34	2.13	0.10
Model 4	–	0.09	–	−	0.14	−	5	1,080.58	2.28	0.09
Model 5	–	–	–	+	0.18	+	7	1,081.35	3.13	0.06
Model 6	–	–	–	+	0.17	−	5	1,082.41	4.10	0.04
Model 7	–	0.13	–	−	–	−	4	1,082.57	4.17	0.37
Model 8	–	0.10	–	+	0.15	+	8	1,083.18	4.79	0.03
Model 9	–	0.13	–	−	–	+	6	1,083.37	4.97	0.02
Model 10	–	–	–	−	–	−	3	1,084.85	6.45	0.01
Model 11	–	0.09	–	+	0.14	−	6	1,084.98	6.58	0.01
Model 12	–	0.14	–	+	–	+	7	1,086.03	7.63	0.01
Model 13	–	–	–	−	–	+	5	1,086.39	7.99	0.01
Model 14	–	–	−0.01	−	0.19	+	7	1,086.59	8.19	0.00
Model 15	–	0.13	–	+	–	−	5	1,086.92	8.53	0.00
Model 16	–	–	−0.01	−	0.18	−	5	1,087.72	9.33	0.00
Model 17	0.00	–	–	−	0.17	−	5	1,088.12	9.72	0.00
Model 18	–	–	–	+	–	−	4	1,088.85	10.45	0.00
Model 19	–	–	−0.01	+	0.19	+	8	1,088.93	10.53	0.00
Model 20	0.00	–	–	−	0.17	+	7	1,089.00	10.61	0.00
Model 21	–	–	–	+	–	+	6	1,089.36	10.96	0.00
Model 22	–	0.09	−0.01	−	0.16	+	8	1,089.48	11.08	0.00
Model 23	–	0.09	−0.01	−	0.15	−	6	1,089.94	11.54	0.00
Model 24	–	–	−0.01	+	0.18	−	6	1,090.31	11.91	0.00
Model 25	0.00	0.09	–	−	0.14	−	6	1,090.44	12.04	0.00
Model 26	0.00	0.10	–	−	0.14	+	8	1,090.69	12.30	0.00
Model 27	–	0.09	−0.01	+	0.16	+	9	1,091.67	13.27	0.00
Model 28	0.00	0.13	–	−	–	−	5	1,092.04	13.64	0.00
Model 29	0.00	–	–	+	0.18	+	8	1,092.15	13.75	0.00
Model 30	0.00	–	–	+	0.17	−	6	1,092.35	13.95	0.00
Model 31	–	0.13	0.00	−	–	−	5	1,092.72	14.33	0.00
Model 32	–	0.08	−0.01	+	0.15	−	7	1,093.23	14.83	0.00
Model 33	0.00	0.13	–	−	–	+	7	1,093.28	14.88	0.00
Model 34	–	0.13	0.00	−	–	+	7	1,093.31	14.91	0.00
Model 35	0.00	0.10	–	+	0.14	+	9	1,093.73	15.33	0.00
Model 36	0.00	–	–	−	–	−	4	1,094.17	15.77	0.00
Model 37	0.00	0.09	–	+	0.14	−	7	1,094.79	16.39	0.00
Model 38	–	–	0.00	−	–	−	4	1,095.30	16.90	0.00
Model 39	–	–	−0.01	−	–	+	6	1,095.32	16.93	0.00
Model 40	–	0.13	−0.01	+	–	+	8	1,095.64	17.24	0.00
Model 41	0.00	–	−0.01	−	0.18	+	8	1,096.09	17.69	0.00
Model 42	0.00	–	−0.01	−	0.18	−	6	1,096.26	17.86	0.00
Model 43	0.00	0.14	–	+	–	+	8	1,096.34	17.94	0.00
Model 44	–	0.12	−0.01	+	–	−	6	1,096.38	17.98	0.00
Model 45	0.00	0.13	–	+	–	−	6	1,096.43	18.03	0.00
Model 46	0.00	–	–	−	–	+	6	1,096.46	18.07	0.00
Model 47	–	–	−0.01	+	–	+	7	1,097.91	19.51	0.00
Model 48	–	–	−0.01	+	–	−	5	1,098.03	19.63	0.00
Model 49	0.00	–	–	+	–	−	5	1,098.31	19.92	0.00
Model 50	0.00	0.09	−0.01	−	0.15	−	7	1,098.45	20.05	0.00
Model 51	0.00	–	−0.01	+	0.18	−	7	1,098.73	20.33	0.00
Model 52	0.00	–	−0.01	+	0.18	+	9	1,098.86	20.46	0.00
Model 53	0.00	0.08	−0.01	−	0.15	+	9	1,098.98	20.59	0.00
Model 54	0.00	–	–	+	–	+	7	1,099.67	21.27	0.00
Model 55	0.00	0.13	−0.01	−	–	−	6	1,101.13	22.74	0.00
Model 56	0.00	0.09	−0.01	+	0.16	+	10	1,101.64	23.24	0.00
Model 57	0.00	0.08	−0.01	+	0.15	−	8	1,101.69	23.29	0.00
Model 58	0.00	0.13	−0.01	−	–	+	8	1,102.72	24.32	0.00
Model 59	0.00	–	0.00	−	–	−	5	1,103.69	25.29	0.00
Model 60	0.00	–	−0.01	−	–	+	7	1,104.69	26.29	0.00
Model 61	0.00	0.12	−0.01	+	–	−	7	1,104.74	26.35	0.00
Model 62	0.00	0.13	−0.01	+	–	+	9	1,105.50	27.10	0.00
Model 63	0.00	–	−0.01	+	–	−	6	1,106.32	27.92	0.00
Model 64	0.00	–	−0.01	+	–	+	8	1,107.63	29.23	0.00
R.I.	–	–	–	−	1	0.47				

Note

95% confidence intervals provided in parentheses.

Abbreviations: −, explanatory variables not included in the model selection; AICc, Akaike's Information Criterion corrected for small sample size; *df*, degrees of freedom; R.I., relative importance; ΔAICc, difference in AICc from the top‐ranked model and model in consideration.
